# AgCu NP Formation by the Ag NP Catalysis of Cu Ions at Room Temperature and Their Antibacterial Efficacy: A Kinetic Study

**DOI:** 10.3390/molecules27206951

**Published:** 2022-10-17

**Authors:** Yujie Tao, Fang Zhou, Kaixin Wang, Dequan Yang, Edward Sacher

**Affiliations:** 1 Solmont Technology Wuxi Co., Ltd., 228 Linghu Blvd, Tian’an Tech Park, A1-602, Xinwu District, Wuxi 214135, China; 2 Division of Natural and Applied Sciences, Duke Kunshan University, Kunshan 215316, China; 3 Hefei Zhonghang Nanotechnology Development Co., Ltd., Gangji Town Industrial Park, Changfeng County, Hefei 231100, China; 4 Regroupement Québécois de Matériaux de Pointe, Département de Génie Physique, Polytechnique Montréal, Case Postale 6079, Succursale Centre-Ville, Montréal, QC H3C 3A7, Canada

**Keywords:** Ag nanoparticles, AgCu nanoalloys, antibacterial efficacies, reaction kinetics

## Abstract

Although a facile route to prepare AgCu nanoalloys (NAs) with enhanced antibacterial efficacy using Ag NP catalysis of Cu ions at elevated temperatures was previously developed, its detailed reaction process is still unclear due to the fast reaction process at higher temperatures. This work found that AgCu NAs can also be synthesized by the same process but at room temperature. AgCu NAs formation kinetics have been studied using UV–Visible spectra and Transmission Electron Microscopy (TEM), where formation includes Cu^2+^ deposition onto the Ag NP surface and Ag^+^ release, reduction, and agglomeration to form new Ag NPs; this is followed by a redistribution of the NA components and coalescence to form larger AgCu NPs. It is found that SPR absorption is linear with time early in the reaction, as expected for both pseudo-first-order (PFO) and pseudo-second-order (PSO) kinetics; neither model is followed subsequently due to contributions from newly formed Ag NPs and AgCu NAs. The antibacterial efficacy of the AgCu NAs thus formed was estimated, with a continuous increase over the whole alloying process, demonstrating the correlation of antibacterial efficacy with the extent of AgCu NA formation and Ag^+^ release.

## 1. Introduction

Ag-based nanoalloys (NAs), especially AgCu, have been of great interest in recent decades due to their enhanced antibacterial efficacies and the reduced amount of silver they use [1,2,3]. The enhanced antibacterial efficacy of AgCu NAs is greater than that of Ag NPs, Cu NPs, and their mixture [4], although the exact mechanism is still unknown [5,6]. AgCu NAs also exhibit less toxicity to human cells. For example, Thakore et al. [7], using the MTT assay method, reported that AgCu NAs prepared using the fruit latex of *Achras sapota Linn* showed lower toxicity to mouse embryonic fibroblast 3T3L1 cells. Długosz et al. [3] recently disclosed that AgCu NAs not only exhibit better antibacterial and antiviral efficacies, but they also showed no genotoxicity compared to Ag or Cu NPs, even at concentrations as high as 500 ppm. Al Tamimi et al. [8] found that Ag NPs showed no toxic effects on healthy cells but were toxic to MCF-7 human breast cancer cells at a concentration of 10 ppm. Ashraf et al. [9] showed that AgCu NAs conjugated with wheat germ agglutinin exhibited toxicity to homologous (MCF-7 and MDA-MB 231) breast cancer cells. Kushwah et al. [10] reported that the biosynthesis of AgCu NAs exhibited enhanced methylene blue photocatalytic and antibacterial efficacies. Bello-Lopez et al. [11] used AgCu NAs deposited onto nonwoven polypropylene for protection against SARS-CoV-2 and found no toxicity for human fibroblasts and keratinocyte cells; additionally, AgCu NAs showed better antioxidant and catalytic performances.

Ameen [12] developed the synthesis of fungus-mediated AgCu NAs that showed enhanced antibacterial efficacy, and their DPPH (α, α-diphenyl-β-picrylhydrazyl) and hydrogen peroxide scavenging activities were high. Kim et al. [13] found that AgCu NAs showed enhanced resistance to oxidation. Gao et al. [14] found that AgCu NAs embedded in Al_2_O_3_ films displayed enhanced nonlinear optical properties. Jin et al. [15] reported that the AgCu dendritic structure showed high catalytic activity for oxygen reduction (i.e., ORR) in an alkaline solution, which improves zinc–air battery performance.

There are several methods to prepare AgCu NAs, including chemical reaction [16,17], laser melting in liquids [18,19], electrochemical fabrication [20], and vacuum sputtering [21]; more detailed information can be found in recent reviews [4,22]. The chemical reduction reaction is simple and easily mass-produced. The main issues of AgCu NA preparation are high antibacterial efficacy, low toxicity, and high stability, which are strongly associated with the physicochemistry of the NAs and their stabilizers [23]. A recent study showed that biosynthesized AgCu NAs might have even better antibacterial efficacy [7,9,24,25,26,27,28]. These studies used plant extracts to synthesize AgCu NAs or bacteria to reduce Ag^+^ and Cu^2+^ and stabilize the AgCu NAs [12,29]. 

Recent studies [5,30] found that AgCu NAs can be synthesized by the Ag NP catalysis of Cu^2+^ at 85 °C, which showed a greatly enhanced antibacterial efficacy. It is believed that the enhanced antibacterial mechanism partly involves Ag^+^ released during AgCu NA formation.

Despite both TEM and UV-Vis spectra having been extensively used to characterize Ag and AgCu NPs [1,2,3,4,5,31], including NPs size and distribution, crystallinity and composition, and aggregation by TEM and its color change by UV-Vis spectra, there is little research on the reaction kinetic process of Ag or AgCu NPs by UV-Vis so far. Using SPR (surface plasma resonance) of Ag NPs’ surface physiochemical data at room temperature may produce useful information if there is a reaction between AgNPs and the Cu ion system. 

In this work, the possibility of AgCu NAs preparation at room temperature was explored, which allowed the exploration of the kinetic process of AgCu formation due to a slow reaction process in lower temperatures. This is the first time that the kinetic process of AgCu formation has been explored.

Both Zone of Inhibition and MIC assay methods have been used to estimate the antibacterial efficacy of AgCu NAs, demonstrating that this enhanced efficacy can be comparable to that attained with elevated temperatures, which are associated with AgCu NAs’ formation. The AgCu NAs’ preparation at room temperature may promise a low-cost manufacturing process after optimization.

## 2. Results

### 2.1. Appearance Change

A typical color change variation with time, for 10 ppm Ag NPs + 100 ppm Cu^2+^, is shown in Figure S1, in the Supplementary Materials, where it can be seen that the color changes from yellow to almost colorless as a function of time. Note that the color decreases with the increasing Cu^2+^ concentration. 

### 2.2. UV-Visible Spectra

**Figure 1:**
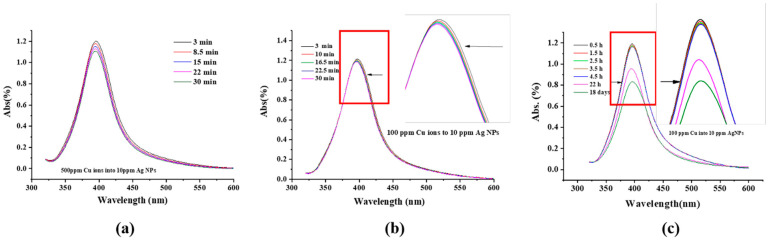
UV-Visible spectra of Cu^2+^ as a function of time at room temperature: (**a**) 500 ppm Cu^2+^ in 10 ppm Ag NPs within 30 min, (**b**) 100 ppm Cu^2+^ in 10 ppm Ag NPs within 30 min, and (**c**) 100 ppm Cu^2+^ in 10 ppm Ag NPs within 18 days.

**Figure 2:**
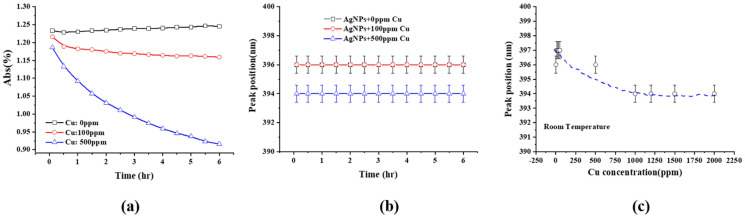
(**a**) SPR peak intensity of 10 ppm Ag NPs + Cu^2+^ as a function of time at room temperature for different Cu ion concentrations, (**b**) SPR peak position of 10 ppm Ag NPs + Cu^2+^ as a function of time for different Cu ion concentrations, and (**c**) SPR peak position (at 10 min) of 10 ppm Ag NPs+ Cu^2+^ as a function of Cu^2+^ concentration.

Representative UV-Visible spectra of 10 ppm Ag NPs + 500 ppm Cu^2+^, evolving as a function of time over 30 min, are found in Figure 1a; the SPR peak, located at ~396 nm, is observed. The SPR evolution of 10 ppm Ag NPs + 100 ppm Cu^2+^ over a longer period of time is found in Figure 1b,c. It shows that the peak intensity decreases over both the early stage (30 min) and a longer time (e.g., 18 days). However, the peak narrows at the higher wavelength side during the early stage (Figure 1a,b) and at the lower wavelength side over the longer time period (Figure 1c). The SPR peak intensity time variation of 10 ppm Ag NPs + Cu^2+^ for different Cu^2+^ concentrations can be found in Figure 2a, and the variations of the peak position with time and Cu^2+^ concentration are shown in Figure 2b,c, respectively. The SPR intensity decreases with time, especially for higher Cu^2+^ concentrations, but its effect on the SPR peak position is minimal. However, increasing the Cu^2+^ concentration leads to a peak shift to lower wavelengths (blue shift, Figure 2c). 

**Figure 3:**
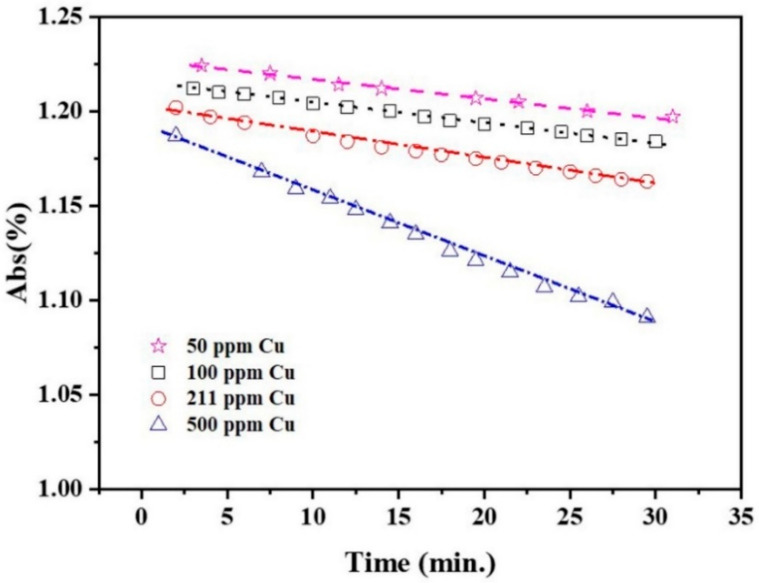
SPR peak intensity of 10 ppm Ag NPs + Cu^2+^ as a function of time at room temperature for different Cu^2+^ concentrations.

Figure 3 shows the SPR peak intensity of 10 ppm Ag NPs + Cu^2+^ as a function of time, for different Cu^2+^ concentrations, during the early stage (e.g., 30 min), indicating that the peak intensity decreases linearly with time for a given Cu^2+^ concentration.

### 2.3. Morphologic Change of Nanoparticles

**Figure 4:**
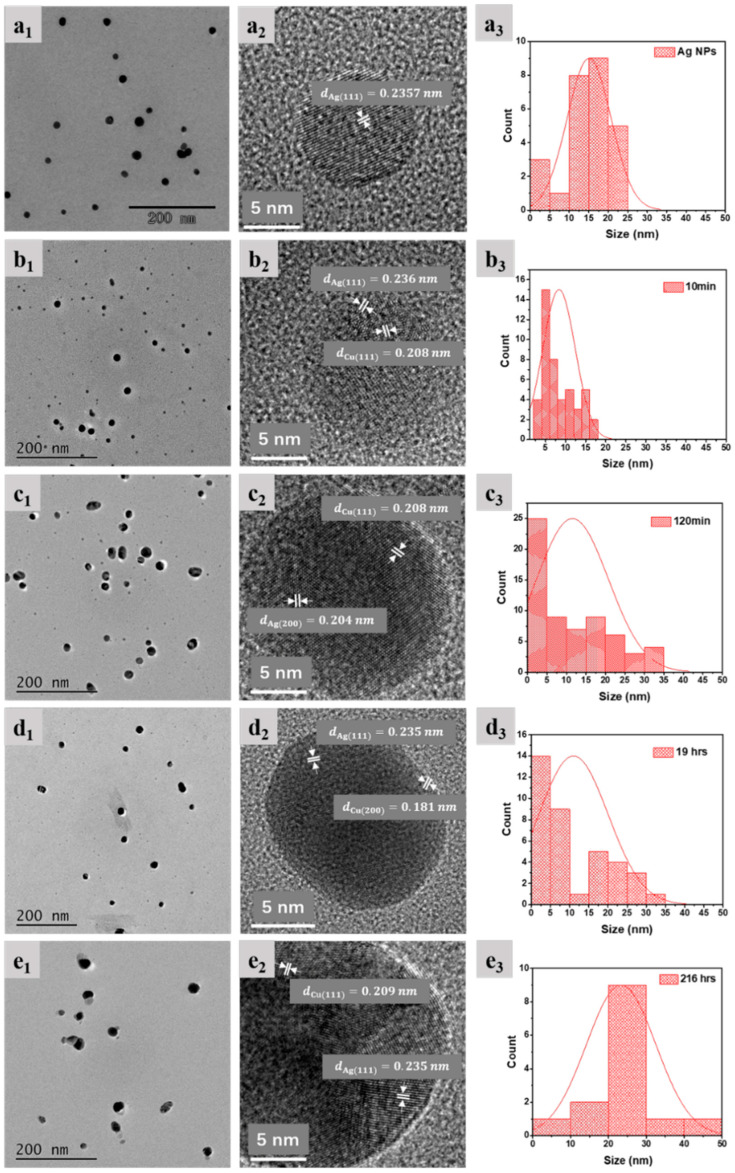
TEM photomicrographs and particle size distributions of 10 ppm Ag NPs + 100 ppm Cu^2+^ as a function of time: (**a**) pure Ag NPs, (**b**) 10 min, (**c**) 120 min, (**d**) 19 h, and (**e**) 9 days.

**Figure 5:**
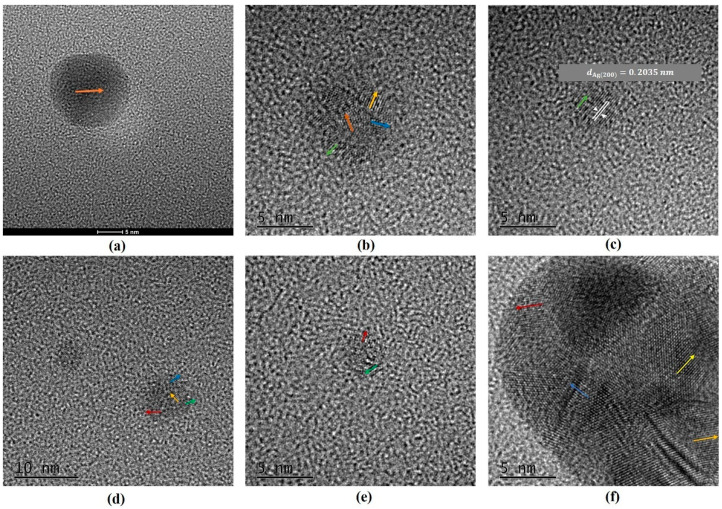
High-resolution TEM of small AgCu NPs from 10 ppm Ag NPs + 100 ppm Cu^2+^ at different times: (**a**) 0 min, (**b**,**c**) 10 min, (**d**) 120 min, (**e**) 19 h, and (**f**) 9 days. The arrows show local lattice orientation with Cu or Ag crystallinity in NPs.

**Figure 6:**
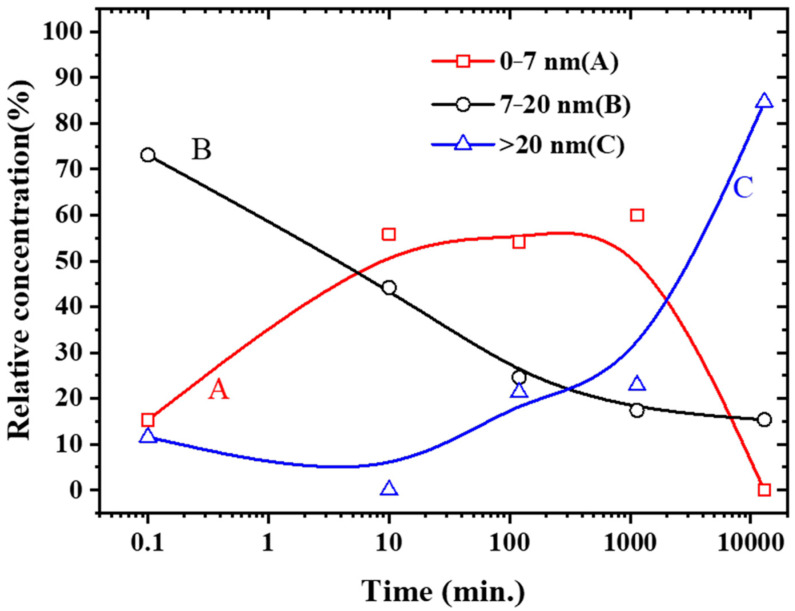
Relative concentration variation of the nanoparticles size distribution for 10 ppm Ag NPs + 100 ppm Cu^2+^ as a function of time. Initial data from pure Ag NPs.

Figure 4 shows TEMs and NP size distributions for 10 ppm Ag + 100 ppm Cu^2+^ as a function of time. One notes many more small NPs (Figure 4b) compared to pure Ag NPs (Figure 4a). It would appear that the average size of the NPs is first reduced before increasing with time (Figure 4c–e). The smaller NPs in Figure 5 appear to be either Ag (e.g., Figure 5c) or, mostly, AgCu (Figure 5b). It is interesting to note that AgCu appears within 10 min (e.g., forming the bimetal structure in Figure 5b). For a detailed analysis of size changes, one can choose to group the sizes thus: from 1 to 7 nm (labeled A), 8 to 20 nm (B), and over 22 nm (C). The A component appears on Cu^2+^ addition, the B component is made up of the original Ag NPs, and the C component appears over time, following Cu^2+^ addition. Their variations with time are seen in Figure 6.

### 2.4. Antibacterial Activity Testing

**Figure 7:**
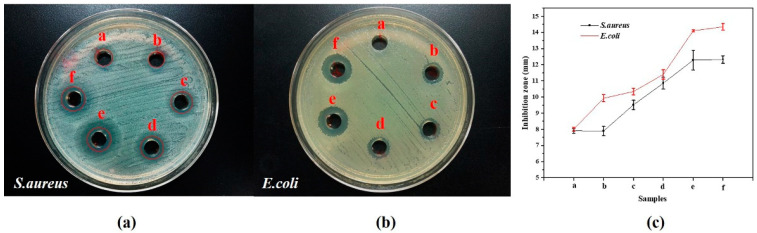
Inhibition zone of 10 ppm Ag NPs + 100 ppm Cu^2+^ as a function of time for (**a**) *S. aureus* and (**b**) *E. coli;* (**c**) Diameter of Inhibition Zone with the samples. a for pure Ag NPs, b for 10min, c for 120 min, d for 24 h, e for 38 days at room temperature and f for 5 h at 85 °C.

Figure 7 and Table S1 in the Supplementary Materials show the antibacterial activity of the 10 ppm AgNPs + 100 ppm Cu^2+^ samples as a function of time, using the agar diffusion method against the two bacterial strains. While they differ significantly between *S. aureus* and *E. coli*, both samples show increased efficacy over time (samples a–e). This is also in good agreement with the MIC assay data in Table S2, which means that a longer time produces lower MICs values. Moreover, it is worth noting that sample e (38 days at room temperature) showed approximately the same antibacterial effects as sample f (at an elevated temperature) against the two strains.

## 3. Discussion

### 3.1. Color Change and UV-Visible Spectra

The color change of the Ag NPs upon adding Cu^2+^, varying with time from yellow to almost colorless after 8 days (Figure S1), is attributed to the reaction of Cu^2+^ with Ag NPs. As in our previous work [5,30], the color of the AgNPs’ dispersion is not only related to the size and density of the NPs, but also to the agglomeration and dispersion of the NPs, so it is difficult to directly judge or determine the cause of color changes over time. However, for a given Ag concentration, the color change can be attributed to the AgCu NAs formation and the associated size change [5,30].

The SPRs of Ag NP dispersions have been well studied. They can be affected by NP shape and size [32,33], as well as the dispersion medium [34,35,36]. They have been used to estimate concentration [37,38,39], invoking the Beer–Lambert law. The spectral evolutions in Figure 1; Figure 2 can be summarized as follows:

(1) The peak intensity decreases with time, which can be caused by either the deposition of an outer layer of Cu or a size reduction in the Ag NPs. Both effectively reduce the Ag NP surface area, and the SPR peak intensity of the Ag NPs is directly proportional to its surface area [40];
(2) The small blue shift of the SPR peak, as the Cu^2+^ concentration increases during the early stage, as seen in Figure 2b, is attributed to the medium refractive index change caused by the change in the dielectric constant [41,42]. Cu^2+^ addition increases the refractive index value of water [43].
(3) The initial SPR peak narrowing at the high wavelength side and the later narrowing at the low wavelength side, in Figure 1a,b, are attributed, respectively, to the NP size decrease and AgCu alloying; alloying NPs will lead to an SPR peak red shift [1,5,44]. This is consistent with the TEM data and will be discussed later.
(4) There is no aggregation of Ag NPs during the reaction and alloying because the appearance of a new peak at 450–600 nm [45,46,47] signifying aggregation was not observed; this is consistent with the TEM photomicrographs.
(5) The dependence of the SPR peak intensity on time and Cu^2+^ concentration (Figure 2a and Figure 3) indicates that the greater the Cu concentration, the greater the SPR intensity decrease. It may indicate that the greater the Cu^2+^ concentration, the faster it is deposited onto the Ag NPs, reducing the uncovered Ag NP area, and diminishing the SPR peak intensity. This will be discussed in Section 3.3.

### 3.2. Morphologic Changes

As shown by the TEMs in Figure 4a and our previous work [5], the Ag NPs are almost monodisperse, most of which are distributed in the 10–25 nm range with an average size of 14 nm; there are few NPs below 7 nm. However, substantial changes occur upon adding Cu^2+^. Figure 4 and Figure 5 show that the deposition of Cu is rapid, and AgCu NAs (about 7 nm in Figure 5b) are produced within 10 min; more small NPs (less than 7 nm) appear afterward (Figure 5 and Figure 6). The small NPs in Figure 4b,c are attributed to the aggregation of Ag^0^ from the reduction of the Ag^+^ released from the Ag-Cu interface [5,6]. Figure 6 indicates that these small NPs remain stabilized for up to 1000 min before disappearing, while the B component decreases and the C component increases. Eventually, the average size of the dispersion increases, consistent with our previous high-temperature AgCu NA synthesis data [5]. 

### 3.3. AgCu NA Formation Kinetics

**Figure 8:**
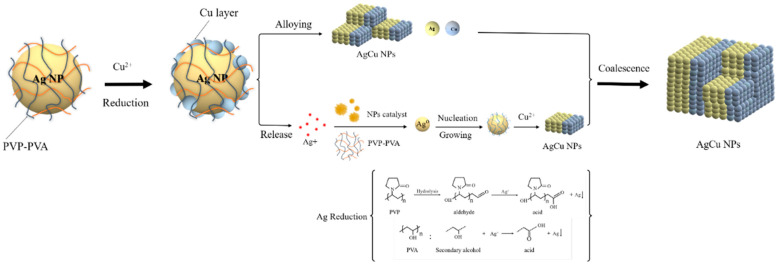
Schematic of AgCu NA formation at room temperature.

Based on the present data, as well as those from the previous higher-temperature work [5], a possible schematic for forming AgCu NAs at room temperature can be found in Figure 8. The Ag NP stabilizers also reduce Cu^2+^ through the catalysis of Ag NPs, depositing Cu^0^ onto the Ag NP surface at room temperature. The Cu-Ag interface thus formed enhances Ag^+^ release, similar to the case at higher temperatures [5]; this continues, with the AgCu NAs becoming small. The released Ag^+^ is easily reduced either by PVP aldehyde chain terminations [48]—similar to what occurs in the Tollens reaction—and/or by the PVA secondary hydroxyl group, forming Ag^0^ followed by Ag agglomeration, leading to the smaller Ag NPs. It must be mentioned that Ag NP catalysis is essential for the reduction of Ag^+^ and Cu^2+^; the presence of Cu^2+^ will suppress Ag ion reduction by PVA [49]. Cu^0^ deposits onto the smaller Ag NP surface because of its higher catalytic activity [50], forming AgCu NAs, followed by coalescence and reorganization to form larger AgCu NAs, consistent with the TEM photomicrographs in Figure 4, Figure 5 and Figure 6. 

The SPR spectral absorbance of Ag NPs is expressed by

(1) $$I=I_{1}-I_{2}+I_{3}$$

$I_{1}$ reflects SPR absorption from the effective surface area of the original Ag NPs (effective surface area = original surface area, treated as a sphere, less that covered by Cu^0^). At the endpoint of the reaction, the effective surface area of the Ag NPs equals zero.

$I_{2}$ reflects the effect of the reduction of the original spherical surface area due to Ag^+^ release, reducing the NP surface area until the Ag disappears.

$I_{3}$ reflects the compensation effect from the newly materialized surface area resulting from the agglomeration of the reduced Ag^0^, the coalescence of the newly formed Ag NPs, and any exposed Ag on the coalescing AgCu NAs.

Cu adsorption on the Ag NP surface is generally considered to fit the pseudo-first-order (PFO) Lagergren equation or the pseudo-second-order (PSO) equation with nonlinear formation [51,52,53]. The pseudo-first-order kinetic equation is

(2) $$q_{t}=q_{e}\left(1-e^{-k_{1}t}\right)$$

and the pseudo-second-order kinetic equation is

(3) $$q_{t}=\frac{k_{2}q_{e}^{2}t}{1+k_{2}q_{e}t}$$

where $q_{t}$ and $q_{e}$ are the sorption capacities at time *t* and at equilibrium, respectively; *k*_1_ and *k*_2_ are the rate constants of the PFO and PSO models, respectively. Considering the Cu deposition to be irreversible, the deposition capacity is equivalent to the surface area covered by Cu^0^. This permits us to modify the PFO Equation (2) to

(4) $$r_{t}=q_{e}e^{-k_{1}t}$$

where $r_{t}$ =$q_{e}-q_{t}$ is the exposed Ag NP surface area, while the PSO Equation (3) can be modified to

(5) $$r_{t}=q_{e}\frac{1}{1+k_{2}q_{e}}$$

The SPR peak intensity is directly related to the uncovered Ag NP surface (effective Ag NP surface area) [40] and is proportional to the effective surface area:

(6) $$I\propto r_{t}$$

Therefore, with Equation (6), Equations (4) and (5) can be simplified to

PFO equation:

(7) $$I_{1}\left(t\right)=I_{0}e^{-k_{1}t}$$

PSO equation:

(8) $$I_{2}\left(t\right)=I_{0}\frac{1}{1+k_{2}q_{e}t}$$

where *I*_0_ is the SPR intensity of Ag NPs before Cu deposition, and I(t) is the SPR intensity at time *t* after adding Cu^2+^.

**Figure 9:**
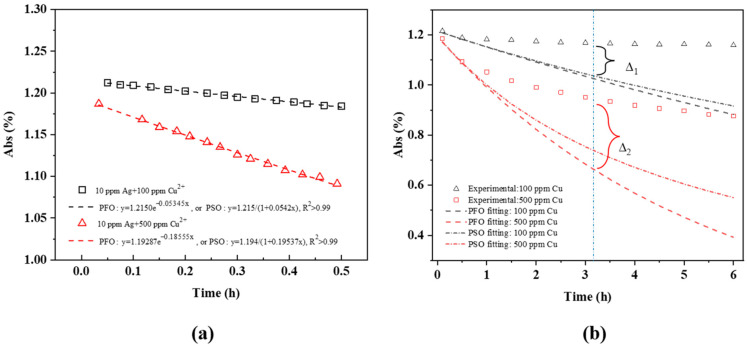
Experimental and theoretical PFO and PFO fits of SPR peak intensity data for 10 ppm Ag NPs + Cu^2+^ as a function of time at room temperature for 100 and 500 ppm Cu^2+^ concentrations; (**a**) within 30 min, (**b**) within 6 h.

Figure 9 shows a comparison of experimental, PFO, and PSO data fits from the SPR intensity variations for 10 ppm AgNPs + 100 ppm Cu^2+^ and 10 ppm Ag NPs + 500 ppm Cu^2+^. During the first 30 min, the Cu^2+^ adsorption onto Ag NPs is accommodated by both PFO and PSO kinetics (Figure 9a), with R^2^ > 0.99, although not afterward, affecting our ability to distinguish between equations. Following the initial 30 min, as seen in Figure 9b, the theoretical predictions for both the PFO and PSO models decrease far more rapidly than in the experiment. This is attributed to contributions from the new Ag NPs and AgCu NAs formed, as illustrated in Figure 8. The Δ_1_ and Δ_2_ differences in Figure 9b indicate that new NP formations now play the dominant role. 

For relatively small *k_i_* and *t*, *k_i_ t*→0, Equations (7) and (8) can be further simplified as linear approximations:

(9) $$I_{1}=I_{0}\left(1-kt\right)\;\mathrm{or}\;I_{1}=I_{0}-Kt$$

This is a linear relationship with time, consistent with Figure 3. 

### 3.4. Antibacterial Efficacy Enhancement of AgCu NAs

Antibacterial efficacy testing, from both the Zone of Inhibition method and MIC test, shows an enhancement of efficacy with time (Figure 7 and Tables S1 and S2). As found in the previous section and illustrated in Figure 8, the AgCu NAs form over time as the Cu is redistributed throughout the Ag NP. This strongly suggests that the enhanced antibacterial efficacy is correlated with the fraction of Ag-Cu interfaces since Ag and Cu are mutually immiscible. That is, aging produces larger NAs with smaller domains and more Ag-Cu interfaces, which implies that the initial step involves bacterial contact with both Ag and Cu surface oxides. The bacteria-NA contact results in enhancing Ag^+^ release [54,55,56,57]; this conclusion is reached by the time dependences of both Ag+ loss and the antibacterial assay. Meanwhile, it is noted that although there are some differences in the details of the two tests (Tables S1 and S2), the general direction of the changes is the same. The difference between the details of Tables S1 and S2 can be attributed to the use of different methods, which are involved in the dilution of an antibacterial agent in a pure liquid process (or liquid–liquid interaction) for MIC testing, while the antibacterial agent is diffused into an agar nutrition process (liquid–solid interaction) for Zone of Inhibition testing. 

It is worth noting that the variation with time of the SPR intensity of AgNPs after adding Cu ions, as shown in Equation (9), can be used directly to estimate Cu ion centration, which will be discussed in a forthcoming paper. Although the process of preparing AgCu NAs at room temperature was indeed time-consuming, it has a lower manufacturing cost. It was also demonstrated that AgCu NAs deposited on fibers had excellent antibacterial efficacy and durability and superior wind resistance compared with Ag ions and AgNPs applied to an air conditioning filter.

## 4. Materials and Methods

### 4.1. Materials

A commercial aqueous dispersion of Ag NPs, 1000 ppm, stabilized by polyvinyl pyrrolidone (PVP) and polyvinyl alcohol (PVA), was provided by Solmont Technologies Co., Ltd. (Wuxi, China). Using both PVA and PVP greatly improves the stability of the Ag NPs, extending their long-term aging time, both at room temperature and 60 °C, enabling their use in commercial applications. Cu(NO_3_)_2_ (Aladdin, AR grade, CAS No 10031-43-3) and Milli Q water were used to synthesize the NAs. All chemicals were used as received. Medium plates were used for bacterial culturing and counting.

Microorganisms: the bacteria used were Gram-positive *Staphylococcus aureus* (ATCC 25923, ATCC 6538) and Gram-negative *Escherichia coli* (ATCC 25922, ATCC 8099).

AgCu NA preparation: the process can be found in our previous article [5]. Briefly, aqueous Cu^2+^ was added to Ag NPs at the appropriate concentration and shaken to mix. All preparations were carried out at room temperature.

### 4.2. Characterization

UV-Visible spectra (723N, Yoke Instruments Co., Ltd., Shanghai, China) were used to record the absorption. High-Resolution Transmission Electron Microscopy (HR-TEM) was carried out on an FEI Tecnai G2 F30 (FEI-Company, Hillsboro, OR, USA).

### 4.3. Antibacterial Assay

The antibacterial efficacies of the NPs were evaluated against *Escherichia coli* and *Staphylococcus aureus*. The inoculums of 0.5 McFarland standards (1.5 × 10^8^ CFU/mL) were maintained in nutrient broth by picking up a single colony from the sub-culture plate [1], then diluted to a concentration of 1.5 × 10^6^ CFU/mL.

The agar diffusion method is considered to be the gold standard of susceptibility testing or the most accurate way to measure the resistance of bacteria to antibiotics [58]. The method was used according to the guidelines of the Clinical and Laboratory Standards Institute (CLSI) [59] with minor modifications. The agar plate surface was inoculated by spreading a volume of the microbial inoculum (1.5 × 10^6^ CFU/mL) over the entire agar surface. Then, wells with a diameter of 8 mm were prepared on the surface of plates, and 50 µL of the antimicrobial agent, at the desired concentration, was introduced into the wells. The Petri dishes were incubated at 37 °C. The antimicrobial agent diffused into the agar and inhibited germination and growth of the test microorganism. The diameters of the inhibition zones were measured by a vernier caliper at least three times [60].

MIC tests were carried out by the Broth dilution method [60]. MIC (Minimum Inhibitory Concentration) is the lowest bacteriostatic concentration of an antibiotic component or agent preventing the visible growth of bacteria. A variety of nanoparticle dilutions was applied to 96-well plates containing bacteria suspension and cultured at 37 °C overnight. Before and after incubation, the MIC was determined by visual observation and validated by turbidity measurements [61]. Each assay was repeated at least 3 times.

## 5. Conclusions

This is the first time SPR has been used to study AgCu NA formation’s kinetic process. It has revealed the AgCu NA formation process—from Cu deposition on the AgNPs’s surface to nucleation, alloying, etc., using UV-Visible spectra and TEM. Smaller AgCu NAs coalesce to form larger NAs. It was not possible to determine whether Cu adsorption onto Ag NPs followed PFO or PSO kinetics since both were linear with time over the first 30 min, and both deviated from the theoretical predictions afterward due to contributions from newly formed Ag NPs and AgCu NAs. It was found that the enhanced antibacterial efficacies for *E. coli* and *S. aureus* using Zone of Inhibition and MIC testing were time-dependent, as was the Ag^+^ release. This work promises a new route to prepare low-cost AgCu NAs on a large scale with enhanced antibacterial efficacy comparable to preparation at elevated temperatures. These AgCu NAs can be further used as antibacterial agents loaded on fibers for application in air condition filters.

## Data Availability

Not applicable.

## Supplementary Materials

The following supporting information can be downloaded at: https://www.mdpi.com/article/10.3390/molecules27206951/s1, Figure S1: The color change of 10 ppm Ag NPs + 100 ppm Cu^2+^ over time; Table S1: Zones of Inhibition of samples of Ag NPs + 100 ppm Cu^2+^, against two strains at various times (a–e) at room temperature, and at 85 °C (f); Table S2. MIC of samples with different time of 10 ppm AgNPs + 100 ppm Cu^2+^ against two strains.

## Sample Availability

Samples of the compounds in this work are available from the authors.

## References

[1] Taner M., Sayar N., Yulug I.G., Suzer S. (2011). Synthesis, characterization and antibacterial investigation of silver–copper nanoalloys. J. Mater. Chem..

[2] Paszkiewicz M., Gołąbiewska A., Rajski Ł., Kowal E., Sajdak A., Zaleska-Medynska A. (2016). Synthesis and characterization of monometallic (Ag, Cu) and bimetallic Ag-Cu particles for antibacterial and antifungal applications. J. Nanomater..

[3] Długosz O., Sochocka M., Ochnik M., Banach M. (2021). Metal and bimetallic nanoparticles: Flow synthesis, bioactivity and toxicity. J. Colloid Interface Sci..

[4] Fan X., Yahia L.H., Sacher E. (2021). Antimicrobial properties of the Ag, Cu nanoparticle system. Biology.

[5] Zhou F., Zhu Y., Yang L., Yang D.-Q., Sacher E. (2022). Ag NP catalysis of Cu ions in the preparation of AgCu NPs and the mechanism of their enhanced antibacterial efficacy. Colloids Surf. A Physicochem. Eng. Asp..

[6] Yang L., Chen L., Chen Y.-C., Kang L., Yu J., Wang Y., Lu C., Mashimo T., Yoshiasa A., Lin C.-H. (2019). Homogeneously alloyed nanoparticles of immiscible Ag–Cu with ultrahigh antibacterial activity. Colloids Surf. B Biointerfaces.

[7] Thakore S.I., Nagar P.S., Jadeja R.N., Thounaojam M., Devkar R.V., Rathore P.S. (2019). Sapota fruit latex mediated synthesis of Ag, Cu mono and bimetallic nanoparticles and their in vitro toxicity studies. Arab. J. Chem..

[8] Al Tamimi S., Ashraf S., Abdulrehman T., Parray A., Mansour S.A., Haik Y., Qadri S. (2020). Synthesis and analysis of silver–copper alloy nanoparticles of different ratios manifest anticancer activity in breast cancer cells. Cancer Nanotechnol..

[9] Ashraf S., Qadri S., Akbar S., Parray A., Haik Y. (2022). Biogenesis of Exosomes Laden with Metallic Silver–Copper Nanoparticles Liaised by Wheat Germ Agglutinin for Targeted Delivery of Therapeutics to Breast Cancer. Adv. Biol..

[10] Kushwah M., Gaur M., Berlina A.N., Arora K. (2019). Biosynthesis of novel Ag@ Cu alloy NPs for enhancement of methylene blue photocatalytic activity and antibacterial activity. Mater. Res. Express.

[11] Bello-Lopez J., Silva-Bermudez P., Prado G., Martínez A., Ibáñez-Cervantes G., Cureño-Díaz M.A., Rocha-Zavaleta L., Manzo-Merino J., Almaguer-Flores A., Ramos-Vilchis C. (2021). Biocide effect against SARS-CoV-2 and ESKAPE pathogens of a noncytotoxic silver–copper nanofilm. Biomed. Mater..

[12] Ameen F. (2022). Optimization of the synthesis of fungus-mediated bi-metallic Ag-Cu nanoparticles. Appl. Sci..

[13] Kim N.R., Shin K., Jung I., Shim M., Lee H.M.J. (2014). Ag–Cu bimetallic nanoparticles with enhanced resistance to oxidation: A combined experimental and theoretical study. J. Phys. Chem. C.

[14] Gao X., Zou C., Zhou H., Yuan C., He J., Luo X. (2020). Enhanced nonlinear optical properties of alloyed AgCu glassy nanoparticles. J. Alloy. Compd..

[15] Jin Y., Chen F. (2015). Facile preparation of Ag-Cu bifunctional electrocatalysts for zinc-air batteries. Electrochim. Acta.

[16] Shin K., Kim D.H., Lee H.M. (2013). Catalytic characteristics of AgCu bimetallic nanoparticles in the oxygen reduction reaction. ChemSusChem.

[17] Reyes-Blas M., Maldonado-Luna N.M., Rivera-Quiñones C.M., Vega-Avila A.L., Roman-Velázquez F.R., Perales-Perez O.J. (2020). Single Step Microwave Assisted Synthesis and Antimicrobial Activity of Silver, Copper and Silver-Copper Nanoparticles. J. Mater. Sci. Chem. Eng..

[18] Shih C.-Y., Chen C., Rehbock C., Tymoczko A., Wiedwald U., Kamp M., Schuermann U., Kienle L., Barcikowski S., Zhigilei L.V. (2021). Limited elemental mixing in nanoparticles generated by ultrashort pulse laser ablation of AgCu bilayer thin films in a liquid environment: Atomistic modeling and experiments. J. Phys. Chem. C.

[19] Swiatkowska-Warkocka Z., Pyatenko A., Krok F., Jany B.R., Marszalek M. (2015). Synthesis of new metastable nanoalloys of immiscible metals with a pulse laser technique. Sci. Rep..

[20] Abdul Salam A., Singaravelan R., Vasanthi P., Bangarusudarsan Alwar S. (2015). Electrochemical fabrication of Ag–Cu nano alloy and its characterization: An investigation. J. Nanostruct. Chem..

[21] Markowska-Szczupak A., Paszkiewicz O., Michalkiewicz B., Kamińska A., Wróbel R.J. (2021). Fabrication of Antibacterial Metal Surfaces Using Magnetron-Sputtering Method. Materials.

[22] Huynh K.-H., Pham X.-H., Kim J., Lee S.H., Chang H., Rho W.-Y., Jun B.-H. (2020). Synthesis, properties, and biological applications of metallic alloy nanoparticles. Int. J. Mol. Sci..

[23] Pfeiffer C., Rehbock C., Hühn D., Carrillo-Carrion C., de Aberasturi D.J., Merk V., Barcikowski S., Parak W.J. (2014). Interaction of colloidal nanoparticles with their local environment: The (ionic) nanoenvironment around nanoparticles is different from bulk and determines the physico-chemical properties of the nanoparticles. J. R. Soc. Interface.

[24] Padilla-Cruz A., Garza-Cervantes J., Vasto-Anzaldo X., García-Rivas G., León-Buitimea A., Morones-Ramírez J. (2021). Synthesis and design of Ag–Fe bimetallic nanoparticles as antimicrobial synergistic combination therapies against clinically relevant pathogens. Sci. Rep..

[25] Al-Haddad J., Alzaabi F., Pal P., Rambabu K., Banat F.J.C.T., Policy E. (2020). Green synthesis of bimetallic copper–silver nanoparticles and their application in catalytic and antibacterial activities. Clean Technol. Environ. Policy.

[26] Escudero-Escribano M., Jensen K.D., Jensen A.W. (2018). Recent advances in bimetallic electrocatalysts for oxygen reduction: Design principles, structure-function relations and active phase elucidation. Curr. Opin. Electrochem..

[27] Zhang Y., Shao Z., Yuan W., Xu H., You X., Liao X. (2021). Green and rapid synthesis of cysteine-directed novel AgCu nanocluster hydrogel with good antibacterial activity. Materialia.

[28] Providence B.A., Chinyere A.A., Ayi A.A., Charles O.O., Elijah T.A., Ayomide H.L. (2018). Green synthesis of silver monometallic and copper-silver bimetallic nanoparticles using Kigelia africana fruit extract and evaluation of their antimicrobial activities. Int. J. Phys. Sci..

[29] Sasireka K.S., Lalitha P. (2021). Biogenic synthesis of bimetallic nanoparticles and their applications. Rev. Inorg. Chem..

[30] Zhu Y., Zhou F., Hu J., Yang L., Yang D.-Q., Sacher E. (2021). A facile route to prepare colorless Ag-Cu nanoparticle dispersions with elevated antibacterial effects. Colloids Surf. A Physicochem. Eng. Asp..

[31] Dou Q., Li Y., Wong K.W., Ng K.M. (2019). Facile synthesis of nearly monodisperse AgCu alloy nanoparticles with synergistic effect against oxidation and electromigration. J. Mater. Res..

[32] Mock J., Barbic M., Smith D., Schultz D., Schultz S. (2002). Shape effects in plasmon resonance of individual colloidal silver nanoparticles. J. Chem. Phys..

[33] Gonzalez A.L., Noguez C., Beranek J., Barnard A.S. (2014). Size, Shape, Stability, and Color of Plasmonic Silver Nanoparticles. J. Phys. Chem. C.

[34] Stockman M.I., Halas N., Huser T. (2005). Plasmonics: Metallic Nanostructures and Their Optical Properties III.

[35] Underwood S., Mulvaney P. (1994). Effect of the solution refractive index on the color of gold colloids. Langmuir.

[36] Yang L., Yang D.-Q., Sacher E. (2022). Colors and surface plasmon resonances of low concentrations of silver nanoparticles in various liquid media. Explor. Mater. Sci. Res..

[37] Paramelle D., Sadovoy A., Gorelik S., Free P., Hobley J., Fernig D.G. (2014). A rapid method to estimate the concentration of citrate capped silver nanoparticles from UV-visible light spectra. Analyst.

[38] Sikder M., Lead J.R., Chandler G.T., Baalousha M. (2018). A rapid approach for measuring silver nanoparticle concentration and dissolution in seawater by UV–Vis. Sci. Total Environ..

[39] Lodeiro P., Achterberg E.P., El-Shahawi M.S. (2017). Detection of silver nanoparticles in seawater at ppb levels using UV–visible spectrophotometry with long path cells. Talanta.

[40] Gómez L.A., de Araújo C.B., Silva A.B., Galembeck A. (2007). Influence of stabilizing agents on the nonlinear susceptibility of silver nanoparticles. JOSA B.

[41] Peng S., McMahon J.M., Schatz G.C., Gray S.K., Sun Y. (2010). Reversing the size-dependence of surface plasmon resonances. Proc. Natl. Acad. Sci. USA.

[42] Mock J.J., Smith D.R., Schultz S. (2003). Local refractive index dependence of plasmon resonance spectra from individual nanoparticles. Nano Lett..

[43] Fen Y.W., Yunus W.M.M. (2011). Characterization of the optical properties of heavy metal ions using surface plasmon resonance technique. Opt. Photonics J..

[44] Zhou M., Wang Z., Sun Q., Wang J., Zhang C., Chen D., Li X. (2019). High-performance Ag–Cu nanoalloy catalyst for the selective catalytic oxidation of ammonia. ACS Appl. Mater. Interfaces.

[45] Ebisawa M., Yamaguchi T., Teranishi Y., Isoda K. (2017). Color modulation of Ag nanoparticle dispersion by light-induced aggregation. IEEJ Trans. Electr. Electron. Eng..

[46] Hu J., Yang L., Zhu Y., Yang D.-Q., Sacher E. (2020). Destabilization of PVA-stabilized Ag NPs: Color changes at low aqueous concentrations, induced by aggregation and coalescence. Mater. Res. Express.

[47] Alqadi M., Abo Noqtah O., Alzoubi F., Alzouby J., Aljarrah K. (2014). pH effect on the aggregation of silver nanoparticles synthesized by chemical reduction. Mater. Sci.-Pol..

[48] Haaf F., Sanner A., Straub F. (1985). Polymers of N-vinylpyrrolidone: Synthesis, characterization and uses. Polym. J..

[49] Dong K. (2023). Cu ions suppression effects of PVA reduction Ag ions and how to overcome it.

[50] Lu Y., Chen W. (2012). Size effect of silver nanoclusters on their catalytic activity for oxygen electro-reduction. J. Power Sources.

[51] Lin Y.-F., Chen H.-W., Chien P.-S., Chiou C.-S., Liu C.-C. (2011). Application of bifunctional magnetic adsorbent to adsorb metal cations and anionic dyes in aqueous solution. J. Hazard. Mater..

[52] Hao Y.-M., Man C., Hu Z.-B. (2010). Effective removal of Cu (II) ions from aqueous solution by amino-functionalized magnetic nanoparticles. J. Hazard. Mater..

[53] Kowanga K.D., Gatebe E., Mauti G.O., Mauti E.M. (2016). Kinetic, sorption isotherms, pseudo-first-order model and pseudo-second-order model studies of Cu (II) and Pb (II) using defatted Moringa oleifera seed powder. J. Phytopharm..

[54] Agnihotri S., Mukherji S., Mukherji S. (2013). Immobilized silver nanoparticles enhance contact killing and show highest efficacy: Elucidation of the mechanism of bactericidal action of silver. Nanoscale.

[55] Bondarenko O., Ivask A., Käkinen A., Kurvet I., Kahru A. (2013). Particle-cell contact enhances antibacterial activity of silver nanoparticles. PLoS ONE.

[56] Li Y., Dong Y., Yang Y., Yu P., Zhang Y., Hu J., Li T., Zhang X., Liu X., Xu Q. (2018). Rational design of silver gradient for studying size effect of silver nanoparticles on contact killing. ACS Biomater. Sci. Eng..

[57] Zhou F. (2022). The enhanced stability and antibacterial efficacy, and the reduced cytotoxicity, of AgCu nanoparticles Incubated with bacteria. Biomater. Adv..

[58] Amsterdam D. (2014). Antibiotics in Laboratory Medicine.

[59] Clinical and Laboratory Standards Institute (2012). Performance Standards for Antimicrobial Disk Susceptibility Tests.

[60] Mb N., Sadiki M., Ibnsouda S.K. (2016). Methods for in vitro evaluating antimicrobial activity: A review. J. Pharm. Anal..

[61] Parvekar P., Palaskar J.N., Metgud S., Maria R., Dutta S. (2020). The minimum inhibitory concentration (MIC) and minimum bactericidal concentration (MBC) of silver nanoparticles against Staphylococcus aureus. Biomater. Investig. Dent..

