# Supplementary material for: AgCu NP Formation by the Ag NP Catalysis of Cu Ions at Room Temperature and Their Antibacterial Efficacy: A Kinetic Study
Source: Molecules. 2022 Oct 17;27(20):6951. doi: 10.3390/molecules27206951 (PMC9607368; doi:10.3390/molecules27206951)
Supplement: Supplementary file 1 [file molecules-27-06951-s001.zip › molecules-1955803-supplementary.pdf]

molecules

MDPI

Supplementary Materials

AgCu NP Formation by the Ag NP Catalysis of Cu Ions at Room Temperature and Their Antibacterial Efficacy: A Kinetic Study

Yujie Tao [1,2], Fang Zhou [1], Kaixin Wang [3], Dequan Yang [1,*] and Edward Sacher [4]

1 Solmont Technology Wuxi Co., Ltd., 228 Linghu Blvd, Tian'an Tech Park, A1-602, Xinwu District, Wuxi 214135, China
2 Division of Natural and Applied Sciences, Duke Kunshan University, Kunshan 215316, China
3 Hefei Zhonghang Nanotechnology Development Co., Ltd., Gangji Town Industrial Park, Changfeng County, Hefei 231100, China
4 Regroupement Québécois de Matériaux de Pointe, Département de Génie Physique, Polytechnique Montréal, Case Postale 6079, Succursale Centre-Ville, Montréal, QC H3C 3A7, Canada
* Correspondence: derry.yang@solmontech.com; Tel.: +86-510-8538-6636

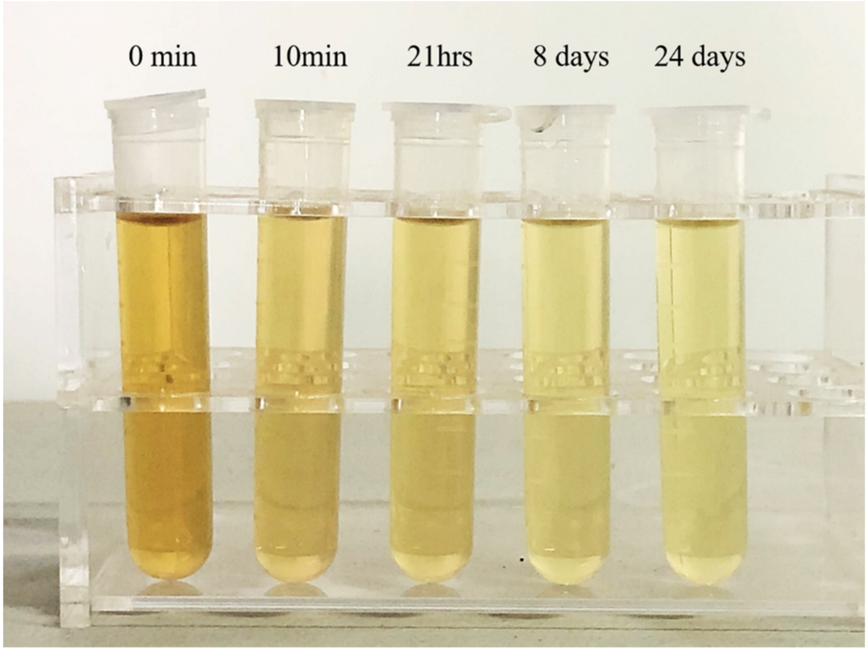

Figure S1. The color change of 10 ppm Ag NPs + 100 ppm Cu^{2+} over time.

Table S1. Zones of Inhibition of samples of Ag NPs+100 ppm Cu^{2+}, against two strains at various times (a–e) at room temperature, and at 85 °C (f).

**Samples (10 ppm Ag NPs+100 ppm Cu)**	**Zone of Inhibition (mm) ± SD (Standard Deviation)**	
	S. aureus	***E. coli***
a (0 min, room temperature)	7.90 ± 0.15	8.01 ± 0.12
b (10 min, room temperature)	7.90 ± 0.29	9.92 ± 0.22
c (120 min, room temperature)	9.51 ± 0.28	10.34 ± 0.21
d (24 hrs, room temperature)	10.86 ± 0.37	11.4 ± 0.30
e (38 days, room temperature)	12.28 ± 0.61	14.11 ± 0.07
f (5 hrs, 85°C)	12.32 ± 0.24	14.35 ± 0.20

Table S2. MIC of samples with different time of 10 ppm AgNPs+100 ppm Cu^{2+} against two strains.

**Samples (10 ppm Ag NPs+ 100 ppm Cu^{2+})**	**MIC(mg/L) against *S. aureus* (ATCC 6538)**		**MIC(mg/L) against *E. coli* (ATCC 8099)**	
	**Concentration of Ag**	**Concentration of Cu**	**C of Ag**	**C of Cu**
a (Ag NPs)	7.8125	--	1.95	--
b (10 min, room temperature)	0.3125	3.125	0.3125	3.125
c (120 min, room temperature)	0.3125	3.125	0.3125	3.125
d (24 hrs, room temperature)	0.3125	3.125	0.15625	1.5625
e (48 hrs, room temperature)	0.3125	3.125	0.15625	1.5625
g (30 days, room temperature)	0.15625	1.5625	0.15625	1.5625
f (5 hrs, 85°C)	0.15625	1.5625	0.15625	1.5625
